# Comparison of serial pancreatic stone protein, C-reactive protein and procalcitonin for the diagnosis of infection and sepsis in critically Ill patients: a multicentre prospective study

**DOI:** 10.1186/s12871-026-03883-z

**Published:** 2026-05-02

**Authors:** Vinod Jaiswal, Kalpana Krishnareddy, Sanjay Nihalani, Nimisha Abdul Majeed, Ribin Raj, Alanduru Nirmala Veenanjani, Prashant Nasa

**Affiliations:** 1 Amina Hospital, ICU, Ajman, United Arab Emirates; 2 Medcare Royal Speciality Hospital, Critical Care, Dubai, United Arab Emirates; 3 Mediclinic Parkview Hospital, Dubai, United Arab Emirates; 4 Critical Care, NMC Royal Khalifa Hospital, Abu Dhabi, United Arab Emirates; 5 Anaesthesia and Critical Care Medicine, Watford General Hospital, West Hertfordshire Teaching Hospitals NHS Trust, Watford, UK; 6 Critical Care Medicine, NMC Speciality Hospital, Dubai, United Arab Emirates; 7 Anaesthesia and Critical Care Medicine, New Cross Hospital, The Royal Wolverhampton NHS Trust, Wolverhampton, UK

**Keywords:** Biomarkers, Infection, Sepsis, Diagnosis, Pancreatic stone protein, C-reactive protein, Procalcitonin

## Abstract

**Background:**

The serial performance of C-reactive protein (CRP), procalcitonin, and emerging biomarker pancreatic stone protein (PSP) was evaluated for the diagnosis of infection and sepsis in patients admitted to the intensive care unit (ICU).

**Methods:**

All consecutive adult patients with suspected infection or sepsis upon their admission to the ICUs of three multi-speciality hospitals in the UAE were enrolled. CRP, procalcitonin, and PSP levels were measured at admission and repeated within 24–48 h. Patients were categorized into infection vs. non-infection, sepsis vs. non-sepsis groups, and into culture-positive and culture-negative subgroups.

**Results:**

A total of 272 ICU patients were analyzed. All biomarkers could be used to distinguish infection with CRP (AUROC 0.77; 95% confidence intervals [CI] 0.70–0.83) and procalcitonin (AUROC 0.75; 95% CI 0.68–0.81) showing fair performance. Moreover, serial monitoring at 24–48 h improved performance, especially for procalcitonin (*p* = 0.04). Among patients with infection, PSP levels were higher in culture-positive compared to culture-negative patients, but the difference did not reach statistical significance (median 229 vs. 142 ng/ml, *p* = 0.05). However, all three biomarkers failed to discriminate sepsis with an AUROC of 0.56 (95% CI 0.49–0.64) for CRP, 0.54 (95% CI 0.46–0.62) for procalcitonin, and 0.58 (95% CI 0.50–0.66) for PSP, respectively. Combining biomarkers improved specificity (93.85%) but with reduced accuracy.

**Conclusion:**

Despite a significant rise in all biomarkers, procalcitonin has overall better performance for diagnosing infections. None of the biomarkers could differentiate sepsis at admission.

**Supplementary Information:**

The online version contains supplementary material available at 10.1186/s12871-026-03883-z.

## Background

Sepsis, defined as a dysregulated response to infection, contributes to considerable morbidity and mortality. According to Global Burden of Disease 2021 estimates, sepsis was responsible for 166 million cases and 21.4 million deaths, accounting for 31.5% of all global deaths [1]. Timely and accurate diagnosis of infection and sepsis is crucial for the prompt initiation of appropriate antimicrobial administration. The risk of worse outcomes with delay in treatment, frequently results in the empirical administration of antibiotics for patients with sepsis. Microbiological cultures, regarded as the reference standard for diagnosing infections and testing antimicrobial sensitivity, are often time-consuming with a poor yield. A meta-analysis of seven studies with a total of 22,625 patients diagnosed with sepsis and septic shock, noted that the culture positivity rate was merely 40.1%. Furthermore, no significant difference in clinical outcomes was observed between patients with positive or negative cultures [2]. In addition, the inappropriate or prolonged use of antimicrobial agents can contribute to antibiotic resistance leading to increased morbidity and mortality. The administration might also lead to adverse effects like *Clostridium difficile-*induced colitis, hypersensitivity reactions, organ toxicity, increased length of stay, and cost [3].

Biomarkers may play a valuable role in helping clinicians with early diagnosis, risk stratification and informed decision-making on the need for antimicrobials [4]. An ideal biomarker should aid in early and accurate diagnosis of sepsis, predict patient deterioration, and be easily accessible and cost-effective [5]. Identifying novel biomarkers for the accurate and early diagnosis of infection or sepsis is ongoing research [5–8]. However, the absence of a definitive “gold-standard” laboratory test for the early identification of infection and sepsis, biomarkers such as C-reactive protein (CRP) and procalcitonin are widely used for diagnosis of infection or sepsis [6, 9].

Pancreatic stone protein (PSP) is a secretory protein produced by pancreatic acinar cells and intestinal Paneth cells that binds to polymorphonuclear cells and activates them. In vitro studies suggest that PSP, through its C-type lectin domain, may activate neutrophils and promote bacterial aggregation in infection and sepsis [10]. Its role as a biomarker has been evaluated to diagnose infection across diverse patient populations and clinical environments [11–16]. Unlike procalcitonin, PSP is not influenced by inflammation caused by non-infectious aetiology [17]. Additionally, PSP is proposed to predict sepsis up to five days earlier than clinical diagnosis [14]. Despite some evidence, PSP needs performance evaluation in multicentre prospective study for diagnosing infection and sepsis in unselected critically ill patients [17, 18]. The study aimed to evaluate the diagnostic performance of serial CRP, procalcitonin and PSP for diagnosing infection and sepsis in patients admitted to the ICU.

## Methods

### Study design and setting

This prospective observational study was conducted in three tertiary care hospitals with mixed medical-surgical ICUs in the United Arab Emirates (UAE), between March 2022 and December 2023. Ethical approval was taken from the Ministry of Health and Prevention Research Ethics Committee (reference number MOHAP/DXB-REC/MMM/No.29/2022) for the Amina Hospital, Ajman, UAE; and Dubai Scientific Research Ethics Committee (DSREC), Dubai Health Authority (reference number DSREC-05/2023_01) for the Mediclinic Parkview Hospital, Dubai, UAE and NMC Specialty Hospital, Dubai, UAE. Informed consent was obtained directly from patients whenever possible. For patients who lacked decision-making capacity due to critical illness, consent was obtained from their legally authorized representatives in accordance with Ethical Committee requirements. All procedures performed in this study involving human participants were conducted in accordance with the principles outlined in the Helsinki Declaration.

### Characteristics of participants

All consecutive critically ill adult patients (aged ≥ 18 years) admitted to the ICU from the emergency department, expected to stay > 48 h, and presenting with signs and symptoms of infection or organ dysfunction were included in the study. The exclusion criteria were suspected or confirmed acute pancreatitis, severe immunosuppression, moribund patients, pregnancy, high-volume blood transfusion, chronic infections such as osteomyelitis, leprosy, brucellosis, malaria, and tuberculosis, and patients transferred from wards or other hospitals with a diagnosis of infection. Moribund patients were defined as those with an expected survival of less than 48 h based on treating clinician assessment at ICU admission, and/or those with severe functional impairment (e.g., Eastern Cooperative Oncology Group performance status ≥ 5) [19].

The study was conducted in three distinct phases throughout the study period, due to the recent COVID-19 pandemic and the availability constraints of PSP assay. Patients were followed until death, discharge from the hospital, or 28 days, whichever is earlier.

### Definitions

Infection was diagnosed based on the treating clinician’s suspicion including history, physical examination, meeting one or more criteria for systemic inflammatory response syndrome (SIRS) and/or microbiologically confirmed infection. Only bacterial and fungal etiologies were primarily considered, while viral and parasitic infections were not systematically assessed due to the lack of routine comprehensive diagnostic testing in our institutions. Sepsis was diagnosed using the third international consensus definition for sepsis and septic shock (Sepsis-3), where organ failure is diagnosed with acute increase in sequential organ failure assessment (SOFA) score by ≥ 2 points [20]. Culture-positive infection or sepsis was the isolation of a pathogenic bacteria or fungal microbe from blood or tissue culture and other appropriate sample.

### Data collection

Data were collected on demographic characteristics, primary diagnosis, SOFA score, and other laboratory tests such as lactates and complete blood count. Plasma levels of PSP, CRP, and PCT were measured on admission to the ICU and within 24–48 h of ICU admission. PSP blood levels were quantitatively measured with point-of-care abioSCOPE^®^ (nanofluidic point-of-care immunoassay; Abionic SA, Switzerland). CRP (particle-enhanced immunoturbidimetric assay) and procalcitonin (chemiluminescent microparticle immunoassay) plasma levels were measured using the Cobas Pure Roche Diagnostics (Switzerland). Additionally, microbiological and radiological data were collected. Patients were categorized as culture-negative and culture-positive infections or sepsis. Furthermore, information was collected on the antimicrobials, including their indication, duration and appropriateness as well as the reason for de-escalation. Biomarker measurements were obtained at admission before antimicrobial administration and repeated after 24–48 h of initiation of antimicrobial therapy.

Patient-centred outcomes, such as the length of hospital stay and 28-day mortality, were also collected. This study was conducted and reported in accordance with the Strengthening the Reporting of Observational Studies in Epidemiology (STROBE) guidelines (Table S1).

### Statistical analysis

The normality of data distribution was assessed using the Kolmogorov–Smirnov test to guide the selection of appropriate descriptive statistics and comparative analyses. Depending on the type of data, mean (standard deviation [SD]), median (interquartile range [IQR]), and frequencies (percentage) were used. Sensitivity, specificity, accuracy, positive predictive value (PPV), and negative predictive value (NPV) were calculated for CRP, procalcitonin, and PSP and their combination. The Mann-Whitney U test was used to compare quantitative variables between the study groups, the chi-square (χ2) test was used for comparing categorical data, and when the expected frequency is less than five, Fisher’s exact test was used. The area under the receiver operating characteristic (AUROC) curve was calculated for the diagnostic performance. The primary objective was to estimate the diagnostic discrimination of PSP for adjudicated infection using the AUROC. Assuming an expected AUROC of 0.90 and an infection prevalence of 54% among included ICU patients, the sample size was determined to achieve a two-sided 95% confidence interval with half-width ± 0.05 for the AUROC [13, 21]. Using a standard AUROC variance approximation (Hanley-McNeil), a total of 160 patients (71 non-infected) were required. To account for missing biomarker data (15–20%) and/or incomplete outcome adjudication (10%), the target sample size was inflated accordingly, resulting in a planned recruitment of approximately 210–225 patients.

We also evaluated the performance of biomarkers and their combination for the diagnosis of infection and sepsis, using the optimal cutoff value obtained from the Youden Index. Optimal cutoff points for each biomarker were identified using the Youden index to provide a standardised, data-driven summary of diagnostic performance. The Youden index was used for exploratory and comparative purposes only, to facilitate uniform comparison across biomarkers within the study cohort, and not to define clinically actionable decision thresholds. A probability value (*p*-value) < 0.05 was considered statistically significant. All statistical calculations were done using Statistical Package for the Social Sciences (SPSS) 21.0 version SPSS Inc., IBM Corp., Armonk, NY, USA.

## Results

**Fig. 1:**
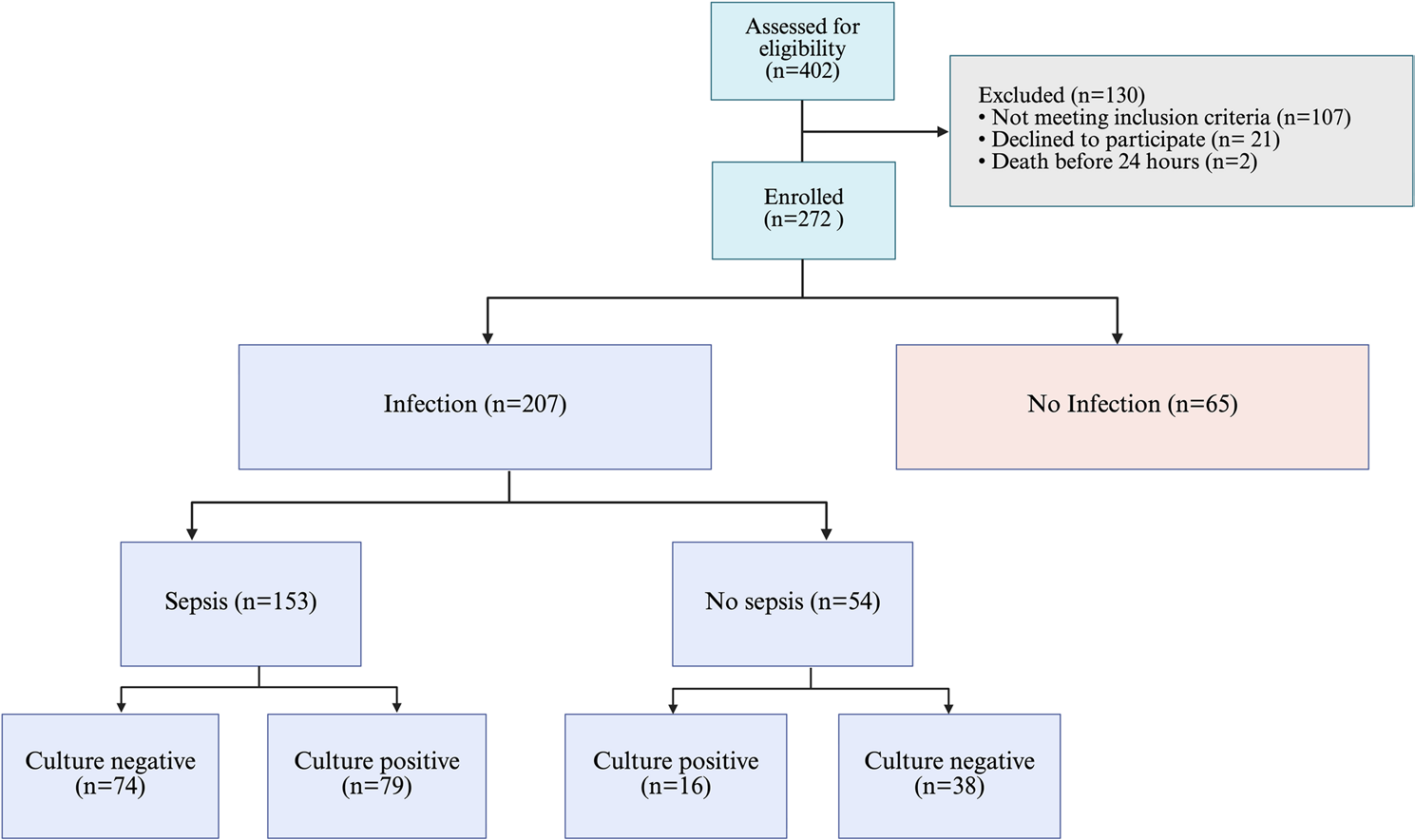
Flow chart of included patients

Of the 272 enrolled patients, 207 (76%) were diagnosed with an infection, and 153 patients had sepsis (53.3%). (Fig. 1**)** The mean age of the cohort was 52.5 (17.5) years, with a predominance of male, 188 (69%). Microbiological cultures were positive in 95 (45.9%) patients. Among patients with sepsis, 79 (38.2%) had culture-positive infection. Patients with sepsis were relatively older with a mean age of 53.1 [17.7] years and had a higher median SOFA score of 5 (3, 12) compared to non-septic patients (1 [0, 5], *p* = 0.001). The mean duration of antibiotics was 8.9 days (4.9), with a relatively longer duration of 10.5 days (3.8) in culture-positive sepsis. The average hospital length of stay was 15 days (26.3). The mortality (10.5%) was higher in septic patients compared to non-septic patients, as well as in culture-negative sepsis (12.2%) compared to culture-positive sepsis (8.9%) (Table 1).

**Table 1:** Demographic and clinical characteristics of subgroup of patients

Variable	Overall infection (*N* = 207)	No Infection (*N* = 65)	Sepsis (*N* = 153)	Culture- positive infection (*N* = 95)	Culture- positive sepsis (*N* = 79)
Age (years)	52.5(17.5)	46.6 (15.7)	53.06 (17.7)	54.33 (18.1)	55.16 (18.1)
Female, n (%)	61 (29.5)	8 (12.3)	49 (32)	29 (30.5)	23 (29.1)
Male, n (%)	146 (70.5)	57 (87.7)	104 (68)	66 (69.5)	56 (70.9)
SOFA day 1 (IQR)	4 (3,7)	2 (3,7)	5 (3,12)	5 (3,7)	5 (3,12)
Duration of antibiotics (days)	8.92 (4.9)	5.03 (3.9)	9.1 (5.2)	10.19 (3.8)	10.49 (3.8)
Length of hospital stay (days)	12.95 (23)	6.78 (4.6)	14.95 (26.3)	18.46 (32.4)	20.49 (35.1)
28-day mortality, n (%)	16 (7.7)	2 (3.1)	16 (10.5)	7 (7.4)	7 (8.9)

*SOFA* Sequential Organ Failure Assessment, *IQR* Interquartile range

*Escherichia coli* (27.3%), *Staphylococcus species* (16.8%) and *Pseudomonas aeruginosa (*14.7%) were the top three isolated pathogens. The predominant source of infection was the respiratory tract (41.2%). Bacteraemia was present in 9.3% of patients. In terms of organ support, 37.1% required vasopressor support, 29.8% required mechanical ventilation, whereas 4.8% required renal replacement therapy.

### Diagnostic performance of biomarkers for the diagnosis of infection

**Fig. 2:**
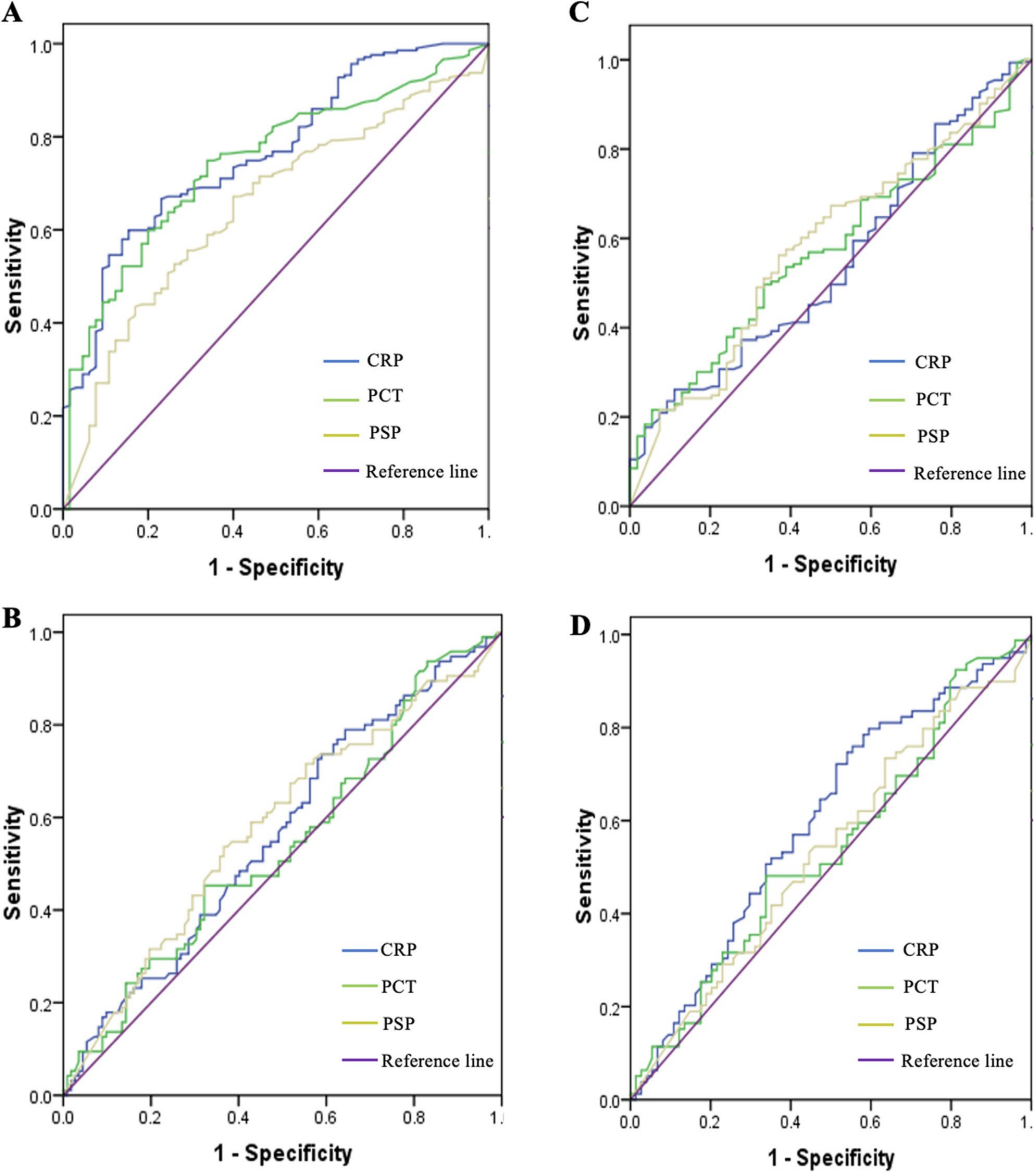
Receiver operating characteristic (ROC) curves of C-Reactive Protein (CRP), Procalcitonin, and Pancreatic Stone Protein (PSP), (**A**) Infection vs non-infection, (**B**) Sepsis vs non-sepsis, (**C**) Culture positive vs culture negative, infection, (**D**) Culture Positive sepsis vs culture negative sepsis

All biomarkers could be used to distinguish infection, with a cutoff 121 mg/ml for CRP, 0.47 ng/ml for procalcitonin, and 111 ng/ml for PSP, respectively. CRP had the highest accuracy with an AUROC of 0.77 (95% confidence intervals [CI] 0.70–0.83) followed by procalcitonin, AUROC 0.75 (95% CI 0.68–0.81). (Fig. 2; Table 2) Furthermore, the accuracy increased with repeating CRP at 24–48 h, AUROC 0.80 (95% CI 0.74–0.86) (Table S2). Among the individual biomarkers, CRP had the highest specificity of 84.6% (95% CI 73.5–92.4) and positive predictive value of 92.5% (95% CI 87.4–95.7), both at baseline and at 24–48 h (sensitivity 84.1% and PPV 93.2%). (Table 3 and S3) Combining all three biomarkers, increased the specificity (92.3%, 95% CI 83–97.5) and PPV 93.8% (95% CI 86.4–97.3), but with a lower sensitivity (36.2%, 95% CI 29.7–43.2).

**Table 2:** Optimal cutoff and AUROC in differentiating infection and sepsis

Biomarkers	Infection (*N* = 207)	No Infection (*n* = 65)				Sepsis (*n* = 153)	No sepsis (*n* = 54) Median			
	Median (IQR)	Median (IQR)	*p*-value	Optimal cutoff	AUROC (95% CI)	Median (IQR)	Median (IQR)	*p*-value	Optimal cutoff	AUROC (95% CI)
CRP (mg/ml)	174 (4–492)	62.9 (1–287)	0.001*	121	0.77 (0.70–0.83)	170 (4–492)	182 (4–345.9)	0.3	286.17	0.55 (0.46– 0.64)
Procalcitonin (ng/ml)	2.2 (0.0–329)	0.25 (0.0–387)	0.001*	0.47	0.75 (0.68–0.81)	2.64 (0.0–329)	1.6 (0.0–329)	0.12	2.67	0.57 (0.49– 0.65)
PSP (ng/ml)	172 (0.8–600)	80 (20–600)	0.001*	111	0.65 (0.58– 0.72)	214 (12.7–600)	122.5 (0.8–600)	0.07	167	0.58 (0.50–0.67)

*AUROC* Area under receiver operating characteristic, *CRP* C-reactive protein, *IQR* Interquartile range, *CI* Confidence interval, *PSP* Pancreatic stone protein, *p-value < 0.05, statistically significant

**Table 3:** Diagnostic performance of biomarkers in distinguishing infection

Biomarker	sensitivity (95% CI)	specificity (95%CI)	accuracy (95% CI)	positive predictive value (95% CI)	negative predictive value (95% CI)
CRP	59.9 (52.9–66.6)	84.6 (73.5–92.4)	65.8 (59.8–71.4)	92.5 (87.4–95.7)	39.9 (35.3–44.6)
Procalcitonin	74.9 (68.4–80.6)	66.2 (53.4–77.4)	72.8 (67.1–78.0)	87.5 (83.3–90.9)	45.3 (38.2–52.6)
PSP	67.2 (60.3–73.5)	60.0 (47.1–72)	65.4 (59.5–71.1)	84.2 (79.6–88.0)	36.5 (30.3–43.1)
PSP + CRP	51.7 (44.7–58.7)	86.2 (75.3–93.5)	59.9 (53.8–65.8)	92.2 (86.5–95.7)	35.9 (32.1–39.9)
PSP+ procalcitonin	52.7 (45.6–59.6)	80.0 (68.2–88.9)	59.2 (53.1–65.1)	89.3 (83.5, 93.3)	34.7 (30.5–39)
PSP + CRP+ procalcitonin	36.2 (29.7–43.2)	92.3 (83.0–97.5)	49.6 (43.5–55.7)	93.8 (86.4–97.3)	31.3 (28.6–34.0)

*CRP* C-reactive protein, *PSP* pancreatic stone protein, *CI* Confidence interval

### Diagnostic performance of biomarkers for the diagnosis of sepsis

When assessing the ability to diagnose sepsis at admission, all three biomarkers failed to discriminate with an AUROC of 0.56 (95% CI 0.49–0.64) for CRP, 0.54 (95% CI 0.46–0.62) for procalcitonin, and 0.58 (95% CI 0.50–0.66) for PSP, respectively (Table 2). Although, combining all three biomarkers increased the specificity for sepsis diagnosis to 96.3% (95% CI 87.3–99.6), overall accuracy was only 36.7% (95% CI 30.1–43.7) (Table 4). Moreover, the procalcitonin rise at 24–48 h was able to distinguish sepsis (2.3 [0–283.4] vs. 1.1 (0.1–53), with a specificity of 94.1% (95% CI 83.8–98.8) (Table S2 and S4).

**Table 4:** Diagnostic performance of biomarkers in diagnosing sepsis

Biomarker	sensitivity (95% CI)	specificity (95% CI)	accuracy (95% CI)	positive predictive value (95% CI)	negative predictive value (95% CI)
CRP	26.1 (19.3–33.9)	88.9 (77.4–95.8)	42.5 (35.7–49.6)	87 (75–93.7)	29.8 (27.1–32.7)
Procalcitonin	49.7 (41.5–57.9)	66.7 (52.5–78.9)	54.1 (47.1–61.03)	80.9 (73.7–86.4)	31.86 (26.8–37.4)
PSP	56.2 (48.0–64.2)	63 (48.7–75.7)	58 (50.9–64.8)	81.1 (74.7–86.22)	33.7 (27.9–40.0)
PSP + CRP	17.7 (12.0–24.6)	94.4 (84.6–98.8)	37.7 (31.1–44.7)	90 (74–96.61)	28.8 (26.9–30.9)
PSP+ procalcitonin	32.0 (24.7–40)	81.5 (68.6–90.8)	44.9 (38.0–52.0)	83.0 (72.8–90)	29.7 (26.4–33.3)
PSP + CRP+ procalcitonin	15.7 (10.3–22.4)	96.3 (87.3–99.6)	36.7 (30.1–43.7)	92.3 (74.5–98)	28.7 (27.0–30.5)

*CRP* C-reactive protein, *PCT* Procalcitonin, *PSP* Pancreatic stone protein, *CI* Confidence interval

**Table 5:** Optimal cutoff and AUROC and in differentiating culture-negative and positive infection and sepsis

	Culture negative infection (*N* = 112)	Culture positive infection (*N* = 95)				Culture-negative sepsis (*N* = 74)	Culture-positive sepsis (*N* = 79)			
	Median (IQR)	Median (IQR)	*p*-value	Optimal cutoff	AUROC (95% CI)	Median (IQR)	Median (IQR)	*p*-value	Optimal cutoff	AUROC (95% CI)
CRP (mg/ml)	164.8 (4–492)	191 (4–464)	0.11	99.5	0.56 (0.49– 0.64)	132 (0–492)	208 (4–464)	0.04*	111	0.60 (051–0.69)
Procalcitonin (ng/ml)	2.2 (0.0–205)	2.2 (0.0–329)	0.31	4.6	0.54 (0.46– 0.62)	2.6 (0.1–205	2.8 (0.0–329);	0.42	4.6	0.54 (0.45– 0.63)
PSP (ng/ml)	142 (0.8–600)	229 (12.7–600)	0.05	217.5	0.58 (0.50–0.66)	181.5 (20–600)	229 (12.7–600)	0.46	212	0.53 (0.44– 0.63)

*AUROC* Area under receiver operating characteristic, *CRP* C-reactive protein, *CI* Confidence interval, *PSP* Pancreatic stone protein

p-value < 0.05, statistically significant

### Differentiating culture-negative and positive infection and sepsis

Among patients with infection, PSP levels were higher in culture-positive than in culture-negative cases, although this difference did not reach statistical difference (median 229 vs. 142 ng/ml, *p* = 0.05), with an AUROC of 0.58 (95% CI 0.50–0.66) and an optimal cutoff of 217.5 ng/ml. In patients with sepsis, CRP discriminated between culture-positive and culture-negative cases (median 208 vs. 132 mg/ml, *p* = 0.04), with an AUROC of 0.60 (95% CI 0.51–0.69) and a cut-off of 111 mg/ml (Table 5). 

### Diagnostic performance of change in biomarkers

Although the biomarker levels increased at 24–48 h from baseline in patients with infection and sepsis, only the procalcitonin change was able to distinguish infection (5.6 [32.6] vs. 5.3 [49.6]; *p* = 0.04) (Table S5). Likewise, in a subgroup analysis of patients receiving appropriate antibiotics, only the change in procalcitonin at 24–48 h reached statistical significance (13.1 [36.3] vs. 7.9 [18.2]; *p* = 0.02 Table S6).

## Discussion

This multicentre observational study evaluated the performance of CRP, procalcitonin, and PSP in diagnosing infection and sepsis in critically ill patients. While all three biomarkers could distinguish patients with infection, only CRP and procalcitonin demonstrated a fair level of diagnostic performance. Furthermore, the diagnostic accuracy for infection improved with repetition of measurements at 24–48 h. None of the biomarkers proved adequate for distinguishing sepsis from patients without sepsis. Notably, the combination of biomarkers increased the likelihood of confirming both infection and sepsis diagnosis.

The findings of this study have several important implications. First, we assessed the real-world performance of PSP in comparison to traditional biomarkers used for diagnosing community-acquired infections and sepsis in critically ill patients. Second, we evaluated the effectiveness of serial measurements of biomarkers to address potential limitations related to their kinetics and relation to inflammation. Lastly, we examined a point-of-care device within a multicentre study, providing insights into the feasibility and generalizability of the results, especially in light of ongoing supply chain challenges of medical consumables, globally.

CRP and procalcitonin are often measured in critically ill patients with clinical diagnosis infection and sepsis [22, 23]. While, the cutoff for CRP in diagnosing infection (121 mg/ml) was higher, the value for procalcitonin (0.47) was consistent with other studies [24–26]. Previous research has indicated that CRP is sensitive in detecting inflammatory conditions, albeit its specificity for infection and sepsis is limited, as elevated levels can occur with non-infectious inflammation [24]. The AUROC values for CRP and procalcitonin in this study align with prior studies, suggesting that CRP alone may be inadequate for infection diagnosis [25]. Procalcitonin is a more accurate biomarker for diagnosing bacterial infection compared to CRP and offers a better balance of sensitivity and specificity [25, 26].

PSP demonstrated the ability to distinguish infection, albeit an inferior performance compared to procalcitonin and CRP. Our results differ from the meta-analyses on the PSP performance [13, 15], which may be attributed to difference in study procedures and patient populations. We used a distinct point-of-care assay, that revealed a higher cutoff for PSP in diagnosing infection compared to the levels (111 vs. 44 ng/ml] reported in meta-analyses [15, 16]. Notably, a similar cutoff has been reported in other studies that used the abioSCOPE point-of-care assay [16, 27]. The predominant cause of infection within our cohort was pneumonia. In another study focussing on ventilator-associated pneumonia, the prediction accuracy of PSP was found to be inadequate [28]. However, the poor performance of PSP in pneumonia needs to be confirmed in future studies.

Furthermore, we evaluated the performance of these biomarkers in a cohort of non-surgical critically ill patients suspected of having community-acquired infection. The metanalyses on PSP have included heterogenous studies with diverse patient population and clinical settings [13, 15]. The absence of trauma and post-operative patients in our study may have contributed to the better performance of CRP and procalcitonin in distinguishing infection, as these non-infectious conditions are usually associated with elevated CRP and procalcitonin [13].

Repeat measurements of biomarkers are superior to single measurements for diagnosing infection, determining its severity and assessing the patient response. PSP levels can rise as early as 5 days compared to procalcitonin (3 days) and CRP (2 days), respectively before a clinical diagnosis of infection [11, 17]. However, monitoring the rate of change and the pattern of response over time can provide a better diagnostic and prognostic information. Additionally, CRP levels usually begin to rise 24 to 48 h after the onset of infection or inflammation and a follow up measurement mitigates the kinetic variability of biomarkers in relation to inflammation and the patients’ condition. This approach also enhances the specificity of the biomarkers in excluding non-infectious cause of infection [10, 11, 18]. While levels of all three biomarkers increased on repeat measurement, only the change in procalcitonin levels (0.3 ng/ml) was statistically significant.

None of the biomarkers individually perform well in distinguishing sepsis from non-septic patients. PSP showed a higher increase, albeit non-statistically significant, in sepsis compared to procalcitonin and CRP, with a median of 214 ng/ml vs. 122.5 ng/ml in the non-sepsis group. However, procalcitonin at 24–48 h, (with a cutoff of 9.1) could differentiate sepsis with an AUROC of 0.62 (95% CI 0.54–0.71). Combining biomarkers improved specificity and PPV, but the sharp decline in sensitivity made this strategy better suited for confirming sepsis, rather than initial screening. The combination of all three biomarkers or only combining PSP and CRP, improved overall performance in diagnosing sepsis, as reported in other studies [13, 15].

To date, most research on PSP has involved small-sized observational studies. Therefore, meta-analyses are merely an aggregation of these studies, with heterogenous patient populations [13, 15], utilising different PSP assays. Our study indicated about the potential limitations of PSP as a standalone diagnostic tool. Further research is required to establish optimal cutoff values and potential clinical applications [18]. This study also highlights that a single threshold based on the Youden index may be suboptimal from a clinical standpoint, suggesting that classification plots may serve as valuable tools to aid clinical decision-making.

We also analysed patients based on their culture results. Overall, the levels of all biomarkers were higher in culture-positive infection and sepsis. However, only CRP plasma levels demonstrated statistically significantly increase in culture-positive sepsis, respectively, implying its potential utility in identifying microbiologically confirmed sepsis when used in conjunction. The AUROC values for all biomarkers, were low (< 0.6), reflecting limited diagnostic precision for culture status.

Our study, has several limitations. First, being an observational multicentre study, confounding factors such as patient comorbidities, ICU admission criteria of individual hospitals, and variations in treatment protocols may have influenced the results. Second, this study primarily evaluated bacterial and fungal infections, and viral or parasitic infections were not systematically assessed due to limited availability of comprehensive diagnostic testing (e.g., viral panels, parasitological investigations). This may limit the generalizability of our findings across all infectious aetiology. Additionally, some culture-negative cases may have represented undiagnosed viral infections, which could partly explain lower procalcitonin and CRP levels and may have attenuated biomarker performance, contributing to lower AUROC values. However, this likely reflects real-world clinical practice in which comprehensive pathogen identification is not always feasible. Third, due to inconsistencies in the supply chain of the diagnostic kits, the study was executed in three phases which may have impacted the validity of the results. Nonetheless, the prospective inclusion of consecutive patients with suspected infection and sepsis helped to mitigate this potential limitation. Fourth, we did not evaluate the impact of antimicrobial and severity stratification on the biomarkers. Additionally, the inclusion criteria restricted patients admitted from the community, which may constrain the generalizability of the findings beyond the study population. Finally, causal relationships between biomarkers and clinical outcomes cannot be firmly established based on the current findings.

## Conclusion

This multicentre prospective observational study evaluated the efficacy of CRP, procalcitonin, and PSP in the diagnosis of infection and sepsis among ICU patients. All three biomarkers proved capable of distinguishing infections in critically ill individuals; however, only CRP and procalcitonin exhibited fair diagnostic accuracy. The diagnostic reliability for identifying infections improved when measurements were repeated after 24–48 h. Nevertheless, none of the biomarkers alone were sufficient to differentiate sepsis from other conditions. While combining multiple biomarkers improved specificity and positive predictive value, the relatively low sensitivity and overall diagnostic accuracy limit their utility as standalone diagnostic tools for identifying infection and sepsis.

## Supplementary Information

Supplementary Material 1: Table S1: STROBE checklist for reporting of observational studies. Table S2: Optimal cut-off and AUROC in differentiating infection and sepsis at 24–48 h. Table S3: Diagnostic performance of biomarkers in distinguishing infection at 24–48 h. Table S4: Diagnostic performance of biomarkers in distinguishing sepsis at 24–48 h. Table S5: Diagnostic performance of change in biomarkers levels between baseline at 24–48 h. Table S6: Diagnostic performance of change in biomarkers levels between baseline at 24–48 h in patients with appropriate antibiotics.

## Data Availability

The datasets used and/or analysed during the current study are available from the corresponding author on reasonable request.

## Abbreviations

CRP: C-reactive protein
PSP: Pancreatic Stone Protein
ICU: Intensive Care Unit
AUROC: Area under the receiver operating characteristic
SOFA: Sequential organ failure assessment
