# Supplementary material for: Comparison of serial pancreatic stone protein, C-reactive protein and procalcitonin for the diagnosis of infection and sepsis in critically Ill patients: a multicentre prospective study
Source: BMC Anesthesiol. 2026 May 2;26:370. doi: 10.1186/s12871-026-03883-z (PMC13281340; doi:10.1186/s12871-026-03883-z)
Supplement: Supplementary file 1 — Supplementary Material 1: Table S1: STROBE checklist for reporting of observational studies. Table S2: Optimal cut-off and AUROC in differentiating infection and sepsis at 24–48 h. Table S3: Diagnostic performance of biomarkers in distinguishing infection at 24–48 h. Table S4: Diagnostic performance of biomarkers in distinguishing sepsis at 24–48 h. Table S5: Diagnostic performance of change in biomarkers levels between baseline at 24–48 h. Table S6: Diagnostic performance of change in biomarkers levels between baseline at 24–48 h in patients with appropriate antibiotics. [file 12871_2026_3883_MOESM1_ESM.docx]

**Comparison of Serial Pancreatic Stone Protein, C-Reactive Protein and Procalcitonin for the Diagnosis of Infection and Sepsis in Critically Ill Patients: A Multicentre Prospective Study**

| **Index** | **Page no** |
| --- | --- |
| Table S1: STROBE checklist for reporting of observational studies | 2 |
| Table S2: Optimal cut-off and AUROC in differentiating infection and sepsis at 24–48 h | 5 |
| Table S3: Diagnostic performance of biomarkers in distinguishing infection at 24–48 h | 5 |
| Table S4: Diagnostic performance of biomarkers in distinguishing sepsis at 24–48 h | 5 |
| Table S5: Diagnostic performance of change in biomarkers levels between baseline at 24–48 h | 6 |
| Table S6: Diagnostic performance of change in biomarkers levels between baseline at 24–48 h in patients with appropriate antibiotics | 6 |

**Table S1:** STROBE Checklist

|  | Item No. | Recommendation | Page  No. | Relevant text from manuscript |
| --- | --- | --- | --- | --- |
| **Title and abstract** | 1 | (*a*) Indicate the study’s design with a commonly used term in the title or the abstract | 1, 2 |  |
|  |  | (*b*) Provide in the abstract an informative and balanced summary of what was done and what was found | 2 |  |
| Introduction | | | |  |
| Background/rationale | 2 | Explain the scientific background and rationale for the investigation being reported | 5,6 |  |
| Objectives | 3 | State specific objectives, including any prespecified hypotheses | 6 |  |
| Methods | | | |  |
| Study design | 4 | Present key elements of study design early in the paper | 7 |  |
| Setting | 5 | Describe the setting, locations, and relevant dates, including periods of recruitment, exposure, follow-up, and data collection | 7 |  |
| Participants | 6 | (*a*) *Cohort study*—Give the eligibility criteria, and the sources and methods of selection of participants. Describe methods of follow-up  *Case-control study*—Give the eligibility criteria, and the sources and methods of case ascertainment and control selection. Give the rationale for the choice of cases and controls  *Cross-sectional study*—Give the eligibility criteria, and the sources and methods of selection of participants | 7 |  |
|  |  | (*b*) *Cohort study*—For matched studies, give matching criteria and number of exposed and unexposed  *Case-control study*—For matched studies, give matching criteria and the number of controls per case |  |  |
| Variables | 7 | Clearly define all outcomes, exposures, predictors, potential confounders, and effect modifiers. Give diagnostic criteria, if applicable | 7, 8 |  |
| Data sources/ measurement | 8* | For each variable of interest, give sources of data and details of methods of assessment (measurement). Describe comparability of assessment methods if there is more than one group | 7, 8 |  |
| Bias | 9 | Describe any efforts to address potential sources of bias | - |  |
| Study size | 10 | Explain how the study size was arrived at | 7 |  |

Continued on next page

| Quantitative variables | 11 | Explain how quantitative variables were handled in the analyses. If applicable, describe which groupings were chosen and why | 8 |  |
| --- | --- | --- | --- | --- |
| Statistical methods | 12 | (*a*) Describe all statistical methods, including those used to control for confounding | 8, 9 |  |
|  |  | (*b*) Describe any methods used to examine subgroups and interactions | 8, 9 |  |
|  |  | (*c*) Explain how missing data were addressed | - |  |
|  |  | (*d*) *Cohort study*—If applicable, explain how loss to follow-up was addressed  *Case-control study*—If applicable, explain how matching of cases and controls was addressed  *Cross-sectional study*—If applicable, describe analytical methods taking account of sampling strategy | 8 |  |
|  |  | (*e*) Describe any sensitivity analyses | - |  |
| Results | | | | |
| Participants | 13* | (a) Report numbers of individuals at each stage of study—eg numbers potentially eligible, examined for eligibility, confirmed eligible, included in the study, completing follow-up, and analysed |  | 10 |
|  |  | (b) Give reasons for non-participation at each stage |  |  |
|  |  | (c) Consider use of a flow diagram |  | Figure 1 |
| Descriptive data | 14* | (a) Give characteristics of study participants (eg demographic, clinical, social) and information on exposures and potential confounders |  | 10, Table 1 |
|  |  | (b) Indicate number of participants with missing data for each variable of interest |  |  |
|  |  | (c) *Cohort study*—Summarise follow-up time (eg, average and total amount) |  |  |
| Outcome data | 15* | *Cohort study*—Report numbers of outcome events or summary measures over time |  | 10, 11 |
|  |  | *Case-control study—*Report numbers in each exposure category, or summary measures of exposure |  |  |
|  |  | *Cross-sectional study—*Report numbers of outcome events or summary measures |  |  |
| Main results | 16 | (*a*) Give unadjusted estimates and, if applicable, confounder-adjusted estimates and their precision (eg, 95% confidence interval). Make clear which confounders were adjusted for and why they were included |  | 10,11 |
|  |  | (*b*) Report category boundaries when continuous variables were categorized |  |  |
|  |  | (*c*) If relevant, consider translating estimates of relative risk into absolute risk for a meaningful time period |  |  |

Continued on next page

| Other analyses | 17 | Report other analyses done—eg analyses of subgroups and interactions, and sensitivity analyses |  |  |
| --- | --- | --- | --- | --- |
| Discussion | | | | |
| Key results | 18 | Summarise key results with reference to study objectives | 12 |  |
| Limitations | 19 | Discuss limitations of the study, taking into account sources of potential bias or imprecision. Discuss both direction and magnitude of any potential bias | 15 |  |
| Interpretation | 20 | Give a cautious overall interpretation of results considering objectives, limitations, multiplicity of analyses, results from similar studies, and other relevant evidence | 12-14 |  |
| Generalisability | 21 | Discuss the generalisability (external validity) of the study results | 12 |  |
| Other information | |  | | |
| Funding | 22 | Give the source of funding and the role of the funders for the present study and, if applicable, for the original study on which the present article is based | 18 |  |

**Table S2: Optimal cut-off and AUROC in differentiating infection and sepsis at 24–48 h**

| **Variable** | **Infection**  **(N=203)** | **No Infection (n=63)** | **p-value** | **Optimal cutoff** | **AUROC (95% CI)** | **Sepsis (n=151)** | **Non- sepsis (n=53)** | **p-value** | **Optimal cutoff** | **AUROC (95% CI)** |
| --- | --- | --- | --- | --- | --- | --- | --- | --- | --- | --- |
| CRP (mg/ml) | 152  (3.1–482) | 44  (5–379) | 0.001* | 101.8 | 0.80 (0.74–0.86) | 153.5 (3.1–482) | 131  (4–469) | 0.27 | 265.5 | 0.55 (0.46–0.64) |
| Procalcitonin  (ng/ml) | 1.81  (0.0–283.4) | 0.20  (0.0–67.54) | 0.001* | 0.7 | 0.79  (0.73–0.85) | 2.3  (0.0–283.4) | 1.1 (0.1–53) | 0.01* | 9.1 | 0.62  (0.54–0.71) |
| PSP (ng/ml) | 155.5  (10–600) | 80  (17–600) | 0.001* | 105 | 0.67  (0.6–0.75) | 170  (20–600) | 124 (10–600) | 0.26 | 126 | 0.55  (0.50–0.64) |

AUROC, area under receiver operating characteristic; CRP, C-reactive protein; IQR, interquartile range; CI, confidence interval; PSP, pancreatic stone protein.

*p-value <0.05, statistically significant

**Table S3: Diagnostic performance of biomarkers in distinguishing infection at 24–48 h**

| **Biomarker** | **sensitivity (95% CI)** | **specificity (95%CI)** | **accuracy**  **(95% CI)** | **positive predictive value (95% CI)** | **negative predictive value (95% CI)** |
| --- | --- | --- | --- | --- | --- |
| CRP (mg/ml) | 67  (60.1–73.4) | 84.1  (72.7– 92.1) | 71.1  (65.2–76.4) | 93.2  (88.4–96.0) | 44.2  (38.8–49.7) |
| Procalcitonin  (ng/ml) | 67.8  (60.9–74.2) | 77.4  (65.0–87.1) | 70.1  (64.2–75.5) | 90.7  (85.9–94.0) | 42.5  (36.7–48.5) |
| PSP (ng/ml) | 67.3  (60.4–73.7) | 61.90  (48.8–73.9) | 66.0  (60– 71.7) | 85.0  (80.3–88.7) | 37.1  (30.9–43.8) |

CRP, C-reactive protein; PSP, pancreatic stone protein; CI, confidence interval.

**Table S3: Diagnostic performance of biomarkers in distinguishing sepsis at 24–48 h**

| **Biomarker** | **sensitivity (95% CI)** | **specificity (95%CI)** | **accuracy**  **(95% CI)** | **positive predictive value (95% CI)** | **negative predictive value (95% CI)** |
| --- | --- | --- | --- | --- | --- |
| CRP (mg/ml) | 20.7  (14.5–28.0) | 90.6  (79.34–96.9) | 38.9  (32.2–46) | 86.1  (71.8–93.8) | 28.7  (26.4–31.25) |
| Procalcitonin  (ng/ml) | 30.5  (23.2–38.5) | 94.1  (83.8–98.8) | 46.5  (39.5–53.7) | 93.9  (83.3–97.9) | 314  (28.7–34.1) |
| PSP (ng/ml) | 60.3  (52–68.1) | 52.9  (38.5–67.1) | 58.4  (51.3–65.3) | 79.1  (73.4–83.9) | 31.0  (24.5–38.4) |

**Table S5: Diagnostic performance of change in biomarkers levels between baseline at 24–48 h**

| **Variable** | **Infection**  **(N=203)** | **No infection (63)** | **p-value** | **Sepsis**  **(N=151)** | **Non-sepsis (51)** | **p-value** |
| --- | --- | --- | --- | --- | --- | --- |
| CRP (mg/ml) | 15.1 (98.7) | 7.7 (81.3) | 0.78 | 17.2 (94.2) | 9 (110.6) | 0.89 |
| Procalcitonin  (ng/ml) | 5.6 (32.6) | 5.3 (49.6) | 0.04 | 7.2  (37.2) | 1 (9.4) | 0.97 |
| PSP (ng/ml) | 8.9 (150.3) | 5.2 (68.5) | 0.90 | 14.8  (156) | -8.5 (131.5) | 0.24 |

*p-value <0.05, statistically significant

**Table S6: Diagnostic performance of change in biomarkers levels between baseline at 24–48 h in patients with appropriate antibiotics**

| **Variable** | **baseline**  **(N=205)** | **At 24-48 h**  **(N=201)** | **Change (Δ)** | **Z** | **p-value** |
| --- | --- | --- | --- | --- | --- |
| CRP (mg/ml) | 159.6 (114.4) | 146.8 (105.9) | 15.1 (98.1) | -0.51 | 0.61 |
| Procalcitonin  (ng/ml) | 13.1 (36.3) | 7.9 (18.2) | 5.5 (29.6) | -2.35 | 0.02 |
| PSP (ng/ml) | 211.9 (182.8) | 194.9 (178.6) | 12.5 (133) | -1.18 | 0.24 |

**p-value <0.05, statistically significant*
